# Development and Pilot Assessment of an AI-Generated Digital Donor Eligibility Screening Tool in Saudi Blood Banks: A Quality Improvement Study

**DOI:** 10.3390/jcm15218548

**Published:** 2026-11-03

**Authors:** Eman M. Mansory, Maha A. Badawi

**Affiliations:** 1 Department of Hematology, Faculty of Medicine, King Abdulaziz University, Jeddah 21589, Saudi Arabia; 2 Hematology Research Unit, King Fahd Medical Research Center, King Abdulaziz University, Jeddah 21589, Saudi Arabia; 3 Blood Transfusion Services Unit, King Abdulaziz University Hospital, Jeddah 21589, Saudi Arabia

**Keywords:** blood donors, artificial intelligence, quality improvement, health information technology, blood banks

## Abstract

**Background:** Manual, paper-based blood donor history questionnaires require blood bank staff to cross-reference lengthy eligibility manuals, which can slow decision-making and introduce inconsistency. Staff-facing digital references have improved workflow efficiency in other healthcare settings, but their use in the Saudi blood donation process has not been explored. **Objective:** To assess the feasibility and staff acceptability of a searchable digital version of the DHQ eligibility manual developed with AI assistance, and to explore its association with perceived screening time and overall process rating. **Methods:** This was a two-center, prospective, pre-post quality improvement pilot conducted between April–August 2026: The Saudi National Donor History Questionnaire (DHQ v1.0) and its 52-item eligibility flowchart were converted into a searchable reference and a digital questionnaire that generates an eligibility output. Baseline and post-implementation staff surveys were administered and compared. **Results:** Twelve responses were obtained at each assessment, the baseline and post-implementation surveys (86% response rate). Perceived time efficiency improved, with 33% of participants reporting the screening process took less than 5 min using the tool, compared with none reporting the same impression before tool use. Overall process rating was unchanged. Perceived tool-specific ratings were favorable across ease of use, speed of retrieval, accuracy, workflow improvement, and satisfaction. Medication-related uncertainty fell from 75.0% to 50.0%. **Conclusions:** An AI-assisted digital adaptation of an existing paper-based donor eligibility manual was feasible and well accepted by staff. More staff reported shorter per-donor assessment times after implementation; however, this exploratory, self-reported finding needs confirmation in larger studies with objective measures.

## 1. Introduction

Accurate and efficient donor eligibility screening is foundational to donor safety, blood safety, and the operational throughput of blood banks [1]. The Donor History Questionnaire (DHQ) is used to assess donor eligibility in reference to national guidelines and blood bank policies. Proper screening minimizes the risk of blood donation by individuals harboring infectious agents, improving the safety of the blood supply [2]. In most centers, screening still relies on a paper-based DHQ form, followed by an interview with blood bank staff to ask additional questions and determine donor eligibility. Donor eligibility decisions are guided by a printed or PDF reference manual (incorporated in blood bank policies). This manual-based workflow places a considerable cognitive and time burden on frontline staff, particularly for less common conditions, medication deferrals, and travel- or exposure-related questions, where flipping through dozens of pages under time pressure increases the risk of delay or inconsistent decision-making. This is especially true in the context of a recent update to eligibility criteria and the Saudi Health Council’s approval of the national DHQ in Saudi Arabia in 2025 [3,4].

Digital clinical decision-support tools have been shown to improve workflow efficiency, standardize decisions, and reduce errors across a range of healthcare settings [5]. Within transfusion medicine, digital and computer-based donor screening approaches have been explored for over two decades and are increasingly relevant as donor eligibility guidelines are updated more frequently and become more complex [6,7]. However, translating a paper manual into a usable digital tool has traditionally required dedicated software development resources, formal informatics support, and multi-month timelines, resources that are rarely available to a hospital blood bank operating a quality improvement (QI) initiative rather than a funded informatics project. Artificial intelligence (AI) can be utilized to facilitate improvement in this matter [8].

Several blood services have transitioned from paper forms to digital pre-donation questionnaires, demonstrating that electronic platforms can improve efficiency while maintaining data quality. The Danish Blood Donor Study showed that a large-scale implementation of a digital questionnaire system improved speed, cost-effectiveness, and flexibility compared to paper-based questionnaires, while still yielding valid data for both clinical and research purposes [9]. Similarly, systems such as the American Red Cross Rapid Pass and online pre-donation questionnaires used by European services enable donors to complete health questionnaires electronically before arrival, streamlining on-site workflows and facilitating rapid updates when criteria change [10].

This paper reports the development process and a pre-post pilot evaluation of a digital screening tool, with three specific aims: (i) to document how an AI language model can be used practically to develop a new clinical support tool; (ii) to assess whether a comparatively simple intervention, a searchable digital reference, with digital DHQ rule based decision support, is associated with changes in staff-perceived workflow; and (iii) to identify future directives from the pilot, including adoption barriers, remaining sources of uncertainty, and implications for scaling this approach to other institutions or guideline updates. No study has evaluated a staff-facing digital conversion of the Saudi National DHQ (v1.0) and its eligibility flowchart to date.

## 2. Materials and Methods

### 2.1. Study Design and Setting

This was a prospective, two-center QI project using a pre-post implementation design, conducted at the blood banks of King Abdulaziz University Hospital (KAUH) and Al Salama Hospital in Jeddah, Saudi Arabia. The project was reviewed and approved as a QI initiative by both institutions’ Research Ethics Committees. Given the QI goal, formal power calculations were not applicable; the project surveyed the entire eligible staff population rather than a sample (*n* = 14). The study took place between April and August 2026.

### 2.2. Participants

All blood bank staff members directly involved in donor eligibility screening at KAUH and Al Salama Hospital blood banks were eligible to participate. They consisted of physicians, nurses, and blood bank/lab technicians and technologists. Twelve responses were obtained at each assessment (12/14 eligible staff, 86%). Because both surveys were fully anonymous, the pre- and post-implementation samples could not be linked at the individual level. However, substantial overlap between the two samples was expected; therefore, they could not be considered independent groups. Informed consent was obtained from participants. No names, employee identifiers, professional experience or role data were captured in the survey instruments used for this pilot.

### 2.3. Development of the Digital Tool

The source material for the tool was the existing, newly developed Saudi National DHQ v1.0 (a 52-item structured donor history questionnaire), together with its accompanying instructional flowchart manual, which specifies the eligibility logic, deferral period, and decision pathway associated with each questionnaire item (e.g., permanent deferral, time-limited/temporary deferral, or further evaluation criteria), and the medication list.

The digital tool was built using Opus/Sonnet 4.5-series, an AI language model, as the primary development aid. The development process consisted of the following steps: (i) Digitization of source content: the 52 questionnaire items and their associated eligibility rules, deferral criteria, and decision flowcharts were structured into a machine-readable format. (ii) AI-assisted conversion: the AI model was used to translate the flowchart logic and free-text eligibility criteria. The version of the Digital Donor Eligibility Assistant evaluated during this pilot was designated Version 1.0, deployed in April 2026, and it included two functionalities. First, an organized, keyword-searchable database that allows the staff to directly clarify the eligibility criteria related to a certain question, medical condition, or medication, instead of searching in the paper manual. Second, an interface that accepts blood bank staff input through a step-by-step digital questionnaire and outputs a clear eligibility decision (eligible, temporarily deferred with duration, or permanently deferred), along with a plain-language justification in both Arabic and English. The tool is based entirely on flowchart logic and eligibility criteria stated in the source manual and does not use AI at runtime to make or suggest eligibility decisions. The questionnaire takes DHQ answers as input, but no donor identifiers (e.g., name, donor serial number, or national identification number). These answers are processed only within the user’s web browser while the page is open. They are not stored, are not sent to any server, and are cleared when the page is closed or reset.

The study team reviewed all AI-assisted content against the original national manual before deployment to ensure decision accuracy. The tool was then tested using completed DHQ forms from 30 donors, purposively selected from real donor eligibility cases in the month before tool deployment. The cases covered the following categories: Accepted donors (7), donors with a single reason for temporary deferral (17), donors with multiple reasons for temporary deferral (3), and Permanent deferral (3). The details of the cases are provided in the Supplementary Materials (File S1-Part A). The reference standard was the eligibility decision recorded on the paper DHQ form, made by the blood bank technician, technologist, or nurse who reviewed the DHQ with the donor, and was then approved by the blood bank physician. The assessor (MB), a blood bank physician, confirmed that each recorded decision was consistent with the DHQ. Each case was then entered into the Digital Donor Eligibility Assistant, and the tool’s output was compared with the recorded decision. No discrepancies were found (30/30 concordant). Donor data were not copied, transmitted, or stored during testing. Records were viewed only by (MB), who was authorised to access donor records at both study sites. This step was included in the ethical approval proposal.

### 2.4. Study Phases

Phase 1—Baseline assessment: An anonymous cross-sectional electronic survey captured participants’ current screening workflow practices, perceived challenges with the paper- or PDF-based manual, estimated time for eligibility decisions, frequency of difficult cases, confidence in final eligibility decisions, and baseline process rating. Participants were also asked which types of questions caused the most uncertainty and what features they would want in a digital tool. See Supplementary (File S1-Part B).

**Figure 1:**
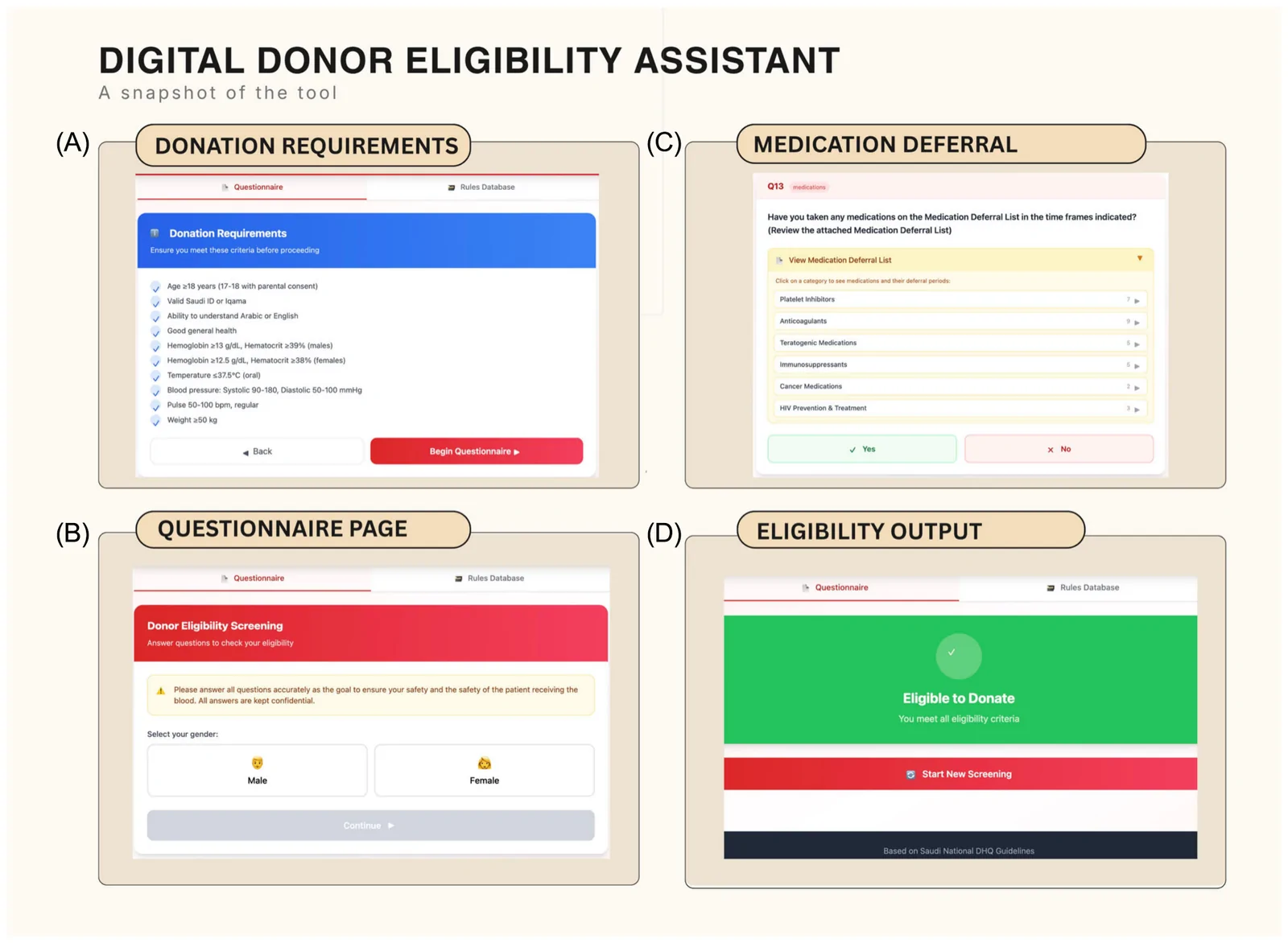
Digital Donor Eligibility Assistant—interface overview. The tool presents (**A**) donor eligibility requirements before screening begins, (**B**) a questionnaire page collecting donor responses, (**C**) a medication deferral question with an expandable, categorized reference list of deferral periods, and (**D**) a plain-language eligibility output summarizing the donor’s screening result. All content is based on the Saudi National Donor History Questionnaire (DHQ v1.0) and its eligibility flowchart manual.

Phase 2—Tool development and deployment: The digital tool was developed as described above and deployed for routine reference use by blood bank staff, with a brief orientation on its search functionality. Please refer to Figure 1 for a screenshot of the tool’s interface.

Phase 3—Post-implementation assessment: After a minimum of 4 weeks of tool availability, staff completed a parallel follow-up electronic survey reassessing workflow efficiency and process ratings using items matched to the baseline survey, plus tool-specific items on ease of use, perceived accuracy, workflow improvement, overall satisfaction, remaining sources of uncertainty, and a single open-ended question on the one change respondents would prioritize. See Supplementary (File S1-Part C).

### 2.5. Outcome Measures

Primary endpoints: (i) change in perceived total eligibility assessment time per donor and (ii) change in overall staff process rating with the donor screening decision-making process (5-point Likert scale).

Secondary endpoints: (i) staff ratings of the tool on a 5-point agreement scale for ease of use; (ii) proportion of staff reporting regular use of the digital tool (adoption rate); and (iii) qualitative categories from open-ended responses regarding remaining uncertainty, and improvement priorities.

### 2.6. Statistical Analysis

Descriptive statistics (frequencies, percentages, means, and standard deviations) were used to summarize categorical and continuous survey items. Both surveys were fully anonymous, and individual responses could not be linked across the two time points, with likely overlap between respondents; therefore, pre- and post-implementation results were compared at the group level descriptively. Free-text responses were few and brief and were analyzed descriptively. One investigator (E.M.) reviewed the responses, identified recurring topics, and grouped similar comments into descriptive categories. These categories were subsequently reviewed by a second investigator (M.B.). The main themes were summarized narratively to reflect common patterns in staff feedback. Statistical analysis was performed using Excel.

### 2.7. Ethical Considerations

This project was conducted and reported as a QI initiative. The ethical approval included all 3 stages of the project. Identifiable donor records were accessed only by an authorized blood bank physician (MB) during tool validation; no identifiers were entered into the tool, recorded, or retained. The survey collected no identifiable staff or donor data. Survey participation was voluntary and anonymous, and informed consent was obtained. Each hospital’s research ethics committee approved the project (Reference No 23–26) at KAUH and (RMEC-ASAH-2026-016) at Al Salama hospital. 

## 3. Results

Twelve responses were obtained at each assessment (12/14, 86% response rate). At baseline, the donor eligibility process was rated positively overall (mean 4.42/5, SD 0.67), and staff reported high confidence in their final eligibility decisions (mean 4.50/5, SD 0.67). Nonetheless, most respondents (8/12, 66.7%) reported that a routine eligibility assessment took 5–10 min per donor, 25.0% (3/12) reported 10–15 min, and 8.3% (1/12) reported more than 15 min; no respondent reported completing an assessment in under 5 min. The steps most frequently identified as the most time-consuming were checking standard operating procedures (SOPs) or flowcharts and medication deferral checks (each cited by 4/12 respondents, 33.3%), followed by clarifying medical history (2/12, 16.7%). Consulting guidelines or a supervisor for clarification was common: 66.7% (8/12) did so “sometimes,” 25.0% (3/12) “rarely,” and 8.3% (1/12) “often.” Printed SOPs/flowcharts (66.7%) and supervisor/colleague consultation (83.3%) were the most frequently used resources at baseline, underscoring the largely manual, paper- and consultation-dependent nature of the pre-implementation workflow.

When asked which question categories caused the most uncertainty (multiple selections permitted), 75.0% (9/12) of respondents cited medication deferral questions and medical conditions (including heart/lung conditions, cancer history, and diabetes) as the leading sources of confusion. These were followed by travel/malaria history (33.3%, 4/12) and brucellosis/raw milk exposure and wounds/infections (each 16.7%, 2/12).

### 3.1. Primary Endpoints: Overall Process Rating and Time Efficiency

After implementing the tool, the overall rating of the donor eligibility process was essentially unchanged from baseline (mean 4.42 [SD 0.67] pre-implementation vs. 4.42 [SD 0.79] post-implementation, *n* = 12 at each time point). The post-implementation evaluation had 7 out of 12 respondents (58.3%) rating the process 5/5.

A clearer signal was seen in perceived assessment time. At baseline, no respondent reported completing an assessment in under 5 min; post-implementation, 33.3% (4/12) reported doing so. The previously modal 5–10 min bracket fell from 66.7% (8/12) to 33.3% (4/12), while 16.7% (2/12) reported 10–15 min, 8.3% (1/12) reported that time “varies widely,” and 8.3% (1/12) reported more than 15 min, matching the baseline proportion in this longest-duration category. Overall, pointing towards a shift in self-reported screening duration.

### 3.2. Secondary Endpoints: Tool Usability, Accuracy, and Workflow Impact

All five tool-specific items, administered only post-implementation, were rated favorably by the 12 respondents, with means ranging from 4.08 to 4.58 out of 5. Workflow improvement again showed the widest spread (SD 0.90), suggesting that the perceived workflow benefit was not uniformly experienced across users. Responses are summarized in Table 1.

Regarding residual sources of uncertainty post-implementation, 58.3% (7/12) of respondents still selected “medical conditions” as a category causing confusion, suggesting that this domain remains an area for future content expansion rather than one the tool has resolved. In contrast, medication-related uncertainty, one of the dominant baseline concerns, dropped to 50.0% from 75%. Sexual risk questions were selected by two respondents (16.7%), while travel/malaria-related questions were selected by one respondent (8.3%), representing a decrease from 33%. Questions related to tattoos, hijama, and piercings were also selected by one respondent (8.3%). Finally, two respondents (16.7%) reported no remaining uncertainty, stating that the tool addressed all their needs.

The reported frequency of active tool use remained modest: one (9.1%) reported using the tool every shift, four (36.4%) reported use in “some shifts” (25–75% of the time), six (54.5%) reported using it “rarely” (<25% of the time), and one skipped this question. For detailed response distributions, see Supplementary File S2.

### 3.3. Qualitative Findings

Baseline (desired features): Free-text responses to “What key features should a screening tool include?” converged on a searchable database, a searchable medication-deferral database with automatic safe/defer/needs-review classification, an auto-updated travel/malaria risk map, tablet- or iPad-based delivery for use during the donor interview that would explain each question for the donors, and, from one respondent, explicit interest in using AI to support the eligibility interview itself.

Post-implementation (improvement priorities): Eight of the twelve respondents provided open-text suggestions; two of these indicated no further changes were needed. Among specific requests, two respondents asked for a donor consent/acknowledgment feature to be added within the tool. One respondent cautioned that the tool “can’t be depended on independently,” i.e., that it should support rather than replace supervisor judgment and institutional SOPs, a comment consistent with the tool’s intended design as a reference resource rather than an autonomous decision-maker. Two respondents raised a related workflow suggestion: one asked that educational materials be presented before the donor questionnaire so that it could be administered directly to donors, and a second proposed a sequential, gated design in which educational materials are completed and signed before the tool proceeds, with the same goal of enabling direct donor administration.

## 4. Discussion

### 4.1. AI as an Accelerator for Piloting New Clinical Tools

This pilot’s central methodological finding is process-related rather than purely quantitative: a simple, functioning, searchable digital decision-support tool was produced from an existing paper manual, was perceived to improve the screening process, and was received positively by staff. This mirrors a broader shift in clinician-led digital health development: recent literature on AI-assisted software development (“vibe coding”) describes how clinicians can now prototype and iterate functional tools directly, addressing workflow problems that are too narrow or low-priority for centralized IT investment, and lowering the barrier to piloting new ideas considerably [11,12,13]. This same literature stresses that lowering the barrier does not remove the need for clinical oversight and complements rather than replaces trained staff [14].

### 4.2. Staff Perceptions of Workflow After Implementation

Despite its simplicity, i.e., a keyword-searchable reference and rule-based questionnaire with no integration with a laboratory or blood establishment information system, the tool was associated with a shift in perceived assessment speed: 33% of post-implementation respondents reported completing a routine assessment in under 5 min, a bracket no baseline respondent selected, while the previously dominant 5- to 10-min bracket fell from 67% to 33%. This is consistent with the basic premise of the intervention: replacing manual page-turning through a paper flowchart with keyword searches plausibly shortens routine lookups, even over a short 4-week pilot.

In contrast, the overall rating of the donor eligibility screening process, the study’s other primary endpoint, was unchanged (4.42/5 at both time points). Several factors could explain this finding. First, the baseline rating was already high, leaving limited room for a ceiling-limited Likert item to move. Second, this global item captures the eligibility decision-making process as a whole, including elements the tool does not touch (e.g., interviewing donors, applying clinical judgment to borderline cases), so a tool that speeds up information retrieval will not necessarily shift a broader process rating. Third, the small sample limits the precision and generalizability of these estimates. Taken together, the data suggest a perceived shift toward shorter assessments and favorable tool-specific ratings despite the lack of evidence in this pilot of an improvement in how staff rate the eligibility process overall.

Medication-related and medical-condition-related questions were equally prominent sources of uncertainty at baseline, each reported by 75% of respondents. Following implementation, the proportion of respondents reporting medication-related uncertainty decreased from 75% to 50%, while travel/malaria-related uncertainty decreased from 33.3% to 8.3%. Both categories therefore showed a descriptive decrease of approximately 25 percentage points. The observed reduction in medication-related uncertainty may reflect the inclusion of the searchable medication list, which was the most frequently requested feature at baseline. However, these findings reflect self-reported perceptions and should be interpreted cautiously given the small sample size. This suggests that a modest, well-targeted digital change, replacing manual page-flipping with a keyword search, can generate a perceived change even in a very short (4-week) pilot, without requiring a more ambitious or costly system integration.

### 4.3. Digitizing the Donation Process

The benefits anticipated from digitizing the donation process are consistent with evidence on computer-assisted donor screening that predates the current era of AI. A methods review of computer-based donor screening concluded that automated computer-assisted interviewing increases elicitation of behaviors associated with transfusion-transmissible infection risk, improves donor and staff satisfaction, and reduces the errors and omissions that frequently accompany traditional interviewing methods [7]. In a randomized crossover trial directly comparing a handheld computerized donor history tool against the standard written questionnaire, the computerized tool identified a blood contact or risk factor for HIV/AIDS or a sexually transmitted infection that the written questionnaire missed in 0.1% to 2.7% of donors. The authors concluded that a self-administered computerized questionnaire may increase risk reporting by blood donors relative to paper-based delivery [15]. Separately, a pilot of an interactive audiovisual computer-based donor interview system found that the approach was well accepted by donors and could prevent many errors [16]. However, no prior local data relevant to this question are available in the Saudi context, underscoring the novelty of the present pilot study as an initial step toward evaluating digital donor-eligibility support in this setting.

More recent evidence extends these findings beyond questionnaire delivery to real-world deferral and eligibility outcomes. In a large-scale implementation at an Austrian blood service, a fully regulation-compliant mobile donation app that digitized the donor questionnaire without disrupting the existing donation workflow reduced the number of deferrals by 31.9% compared to the conventional paper questionnaire, reaching 45,000 users within 18 months of launch [6]. Complementing this, a German blood service found that simply allowing donors to complete the donor history questionnaire at home before their donation visit, rather than under time pressure on-site, reduced the rate of ineligible donors by 27% (from 7.1% to 5.2%), with the large majority of surveyed donors reporting that they had reviewed the questionnaire in advance or were already familiar with its content from previous donations [17]. Together, these two studies suggest that both the format of questionnaire delivery and the timing and setting in which it is completed can independently improve donor eligibility outcomes, reinforcing the rationale for a more fully developed, donor-facing extension of the present tool.

### 4.4. Other Observations from the Pilot

Baseline responses about desired tool features (a searchable medication database with automatic safe/defer/review classification and an auto-updated travel/malaria risk map) directly informed which content areas were prioritized during development, with medications receiving the most development attention, supporting the value of a brief front-end survey in steering an AI-assisted build. By contrast, persistent uncertainty clustered around broader medical-condition questions (cancer, heart/lung disease, diabetes). This likely reflects genuine clinical complexity and physician-level judgment calls rather than a simple content gap, and may be better addressed through case-based training or a clearer escalation pathway than through additional searchable text alone. A notable and somewhat counterintuitive finding was the gap between high satisfaction and usability ratings and modest reported usage frequency, a well-established phenomenon in clinical decision support literature [18]. Plausible explanations include routine, low-ambiguity cases not requiring a lookup at all, staff relying on memory for frequently encountered scenarios, or the 4-week window being too short to establish a consistent habit. This gap between attitude and behavior is itself a useful pilot finding and a natural target for a longer follow-up period. More broadly, the approach demonstrates a template that could plausibly be extended to other donor eligibility updates (for instance, when national guidelines are revised) or adapted by other blood banks facing similar resource constraints.

### 4.5. Limitations

This pilot has several limitations that should inform the interpretation of its findings. The validation process of the tool was limited by the fact that the assessor was neither independent nor blinded, and the 30 cases did not cover every rule or rare decision pathway. The pilot was conducted at two centers with a small sample, limiting precision and generalizability. The anonymous, unpaired pre-post design precludes individual-level change analysis and may under- or overstate the true effect size. The short, 4-week follow-up period may not capture longer-term adoption patterns, and the modest, inconsistent usage frequency observed here may reflect this narrow window rather than a stable adoption ceiling. Outcomes relied on self-reported perception rather than objective time-motion measurement of screening duration or error rates. No professional experience or role data were collected; therefore, subgroup differences by seniority or position could not be examined. Finally, the tool’s clinical content, although reviewed by the study team, was AI-assisted in its generation and requires ongoing, version-controlled clinical governance as guidelines change.

### 4.6. Future Directions

Building on this pilot, several concrete steps could strengthen both the evaluation process and the tool. Methodologically, larger multi-center evaluations with objective time-motion measurements, paired pre-post designs where anonymity permits linkage (e.g., via non-identifying codes), and longer follow-up would provide a more robust evidence base than this two-center pilot allows.

For the tool itself, the next stage of development would move from a searchable reference toward a more integrated system: a structured database in place of the current content, digital donor consent capture, and self-administered delivery of the DHQ with in-line explanations for each question. After adequate validation and approvals, integration with the laboratory/blood establishment information system would further reduce duplicate data entry and allow eligibility determinations to be captured directly within the donor’s record. Such a system could simplify the donation experience for both donors and staff and reduce human error, an effect that the present pilot’s design does not allow us to measure directly.

## 5. Conclusions

This pilot demonstrates that frontline clinical staff can use an AI model to convert an existing paper-based donor eligibility manual into a functioning, searchable digital reference tool and decision support. In a small, two-center sample, this comparatively simple intervention was associated with a shift toward faster perceived per-donor assessment times and fewer staff reporting uncertainty about medications and travel questions. These differences are exploratory and self-reported. Staff also rated the tool favorably for ease of use, perceived accuracy, and satisfaction. These findings support the feasibility and staff acceptability of AI-assisted prototyping for blood bank QI and justify a larger, multi-center evaluation with objective outcome measures and longer follow-up.

## Figures and Tables

**Table 1:** Blood bank staff impressions on tool usability, accuracy, and workflow impact.

Survey Item (Post-Implementation Only, *n* = 12)	Mean ± SD
The tool is easy to use and navigate	4.58 ± 0.51
The tool helps find eligibility information quickly	4.42 ± 0.79
The information in the tool is accurate and reliable	4.42 ± 0.79
The tool has improved my workflow compared to before	4.08 ± 0.90
Overall, I am satisfied with the tool	4.42 ± 0.67

## Data Availability

The data supporting the findings of this study are available from the corresponding author upon reasonable request.

## Supplementary Materials

The following supporting information can be downloaded at: https://www.mdpi.com/article/10.3390/jcm15218548/s1, File S1. Part A. Validation Cases; Part B. Baseline survey: Donor Eligibility Workflow Survey; Part C. Post-implementation survey: Donor Eligibility Digital Assistant Evaluation. File S2. Survey response distributions for close-ended questions. Part A. Baseline survey (*n* = 12); Part B. Post-implementation survey (*n* = 12).
