# Supplementary material for: Development and Pilot Assessment of an AI-Generated Digital Donor Eligibility Screening Tool in Saudi Blood Banks: A Quality Improvement Study
Source: J Clin Med. 2026 Nov 3;15(21):8548. doi: 10.3390/jcm15218548 (PMC13764147; doi:10.3390/jcm15218548)
Supplement: Supplementary file 1 [file jcm-15-08548-s001.zip › Supplementary File S1.pdf]

Supplementary File S1

Part A. Validation Cases:

	Input	Eligibility decision (expected)	Observed output
1	Man. Answered no to all questions causing deferral	No deferral	No deferral
2	Man. Using inhalers for asthma.	No deferral	No deferral
3	Man. Received influenza vaccine 1 week ago	No deferral	No deferral
4	Man. Returned from Egypt 10 days ago.	No deferral	No deferral
5	Man. Returned from Dubai 40 days ago.	No deferral	No deferral
6	Man. Dental cleaning 4 days ago.	No deferral	No deferral
7	Woman. Answered no to all questions causing deferral	No deferral	No deferral
8	Woman. Miscarriage 4 months ago.	Temporary deferral for 9 months post-delivery/miscarriage.	Temporary deferral for 9 months post-delivery/miscarriage.
9	Man. Dental fillings on the same day.	Temporary deferral for 24 hours from time of dental visit.	Temporary deferral for 24 hours from time of dental visit.
10	Man. Diabetes. Diabetic ketoacidosis 10 weeks ago.	Temporary deferral for 3 months from date of episode	Temporary deferral for 3 months from date of episode
11	Woman. On warfarin.	Temporary deferral for 7 days from last dose	Temporary deferral for 7 days from last dose
12	Man. On mycophenolate mofetil	Temporary deferral for 6 weeks from last dose	Temporary deferral for 6 weeks from last dose
13	Man. On isotretinoin for acne.	Temporary deferral for 1 month from last dose	Temporary deferral for 1 month from last dose
14	Man. Antibiotics for respiratory tract infection 2 weeks ago.	Temporary deferral for 1 month from last dose	Temporary deferral for 1 month from last dose
15	Man. On aspirin.	Temporary deferral for 2 days from last dose.	Temporary deferral for 2 days from last dose.
16	Woman. Husband has hepatitis C virus.	Temporary deferral for 6 months from last sexual contact and living together.	Temporary deferral for 6 months from last sexual contact and living together.
17	Woman. Needle stick injury 1 months ago.	Temporary deferral for 6 months from date of injury	Temporary deferral for 6 months from date of injury
18	Man. Had a blood transfusion 2 months ago.	Temporary deferral for 6 months from date of transfusion.	Temporary deferral for 6 months from date of transfusion.
19	Man. Dengue virus infection 3 months ago.	Temporary deferral for 4 months from date of recovery	Temporary deferral for 4 months from date of recovery
20	Woman. Body piercing 1 week ago.	Temporary deferral for 6 months from date of procedure.	Temporary deferral for 6 months from date of procedure.
21	Man. Raw milk consumption 2 days before.	Temporary deferral for 2 months.	Temporary deferral for 2 months.
22	Man. History of hepatitis C.	Permanent deferral.	Permanent deferral.
23	Man. Treated for syphilis 1 month ago. Now on isotretinoin.	Temporary deferral for 3 months post treatment for syphilis and 1 month from last dose of isotretinoin.	Temporary deferral for 3 months post treatment for syphilis and 1 month from last dose of isotretinoin.
24	Man. Donated blood 7 weeks ago.	Temporary deferral for 8 weeks from last whole blood donation.	Temporary deferral for 8 weeks from last whole blood donation.
25	Man. Donated blood 6 weeks ago and had a tattoo 1 month ago.	Temporary deferral for 8 weeks post whole blood donation and 6 months from date of procedure.	Temporary deferral for 8 weeks post whole blood donation and 6 months from date of procedure.
26	Man. On antibiotics and had hijama last month.	Temporary deferral for 1 month after antibiotic use and 6 months after hijama.	Temporary deferral for 1 month after antibiotic use and 6 months after hijama.
27	Man. Lived in India until last year.	Temporary deferral for 3 years.	Temporary deferral for 3 years.
28	Man. Previous history of malaria	Temporary deferral for 3 years from recovery.	Temporary deferral for 3 years from recovery.
29	Man. Previous history of brucellosis.	Permanent deferral.	Permanent deferral.
30	Man. History of hepatitis B. Raw milk ingestion 1 week ago.	Permanent deferral.	Permanent deferral.

Part B. Baseline survey: Donor Eligibility Workflow Survey

B1. How would you rate the current donor eligibility process overall? **(matched to Q P2)**

Response format: 5-point scale, single response

- ☐ 1 = Very difficult
- ☐ 2
- ☐ 3
- ☐ 4
- ☐ 5 = Very efficient

B2. Which parts of the eligibility process take the most time?

Response format: Single response

- ☐ Reviewing DHQ responses
- ☐ Medication deferral checks
- ☐ Travel/malaria questions
- ☐ Sexual risk questions
- ☐ Clarifying medical history
- ☐ Checking SOPs or flowcharts
- ☐ Communicating reasons for deferral
- ☐ Documentation/data entry
- ☐ Other (short answer)

B3. On average, how long does the eligibility assessment take per donor? **(matched to Q P3)**

Response format: Single response

- ☐ <5 minutes
- ☐ 5–10 minutes
- ☐ 10–15 minutes
- ☐ >15 minutes
- ☐ It varies widely

B4. What are the biggest challenges you face during donor eligibility assessment?

يمكنك كتابة الإجابة باللغة العربية أو الإنجليزية، حسب تفضيلك

Response format: Free text (short answer)

B5. Which types of questions cause the most uncertainty or confusion? **(matched to Q P4)**

Response format: Multiple response (check all that apply)

- ☐ Medication deferral list
- ☐ Medical conditions
- ☐ Travel/malaria
- ☐ Wounds/ infection
- ☐ Brucellosis / raw milk
- ☐ Pregnancy/breastfeeding
- ☐ Tattoos, piercing, hijama
- ☐ HIV/sexual risk
- ☐ Other: _________

B6. How often do you need to consult guidelines or supervisors for clarification?

Response format: Single response

- ☐ Never
- ☐ Rarely
- ☐ Sometimes
- ☐ Often
- ☐ Always

B7. On a scale of 1 to 5, how confident are you in the final eligibility decision after completing the current assessment process?

Response format: 5-point scale, single response

- ☐ 1 = Not at all confident
- ☐ 2
- ☐ 3
- ☐ 4
- ☐ 5 = Completely confident

B8. What tools or resources do you currently use most frequently during the eligibility assessment (e.g., printed charts, digital database, supervisor)?

Response format: Multiple response

- ☐ Printed SOPs/Flowcharts
- ☐ Internal Digital Database/System
- ☐ Physical Medication Deferral List
- ☐ Online Resources (e.g., external websites)
- ☐ Supervisor/Colleague Consultation
- ☐ None (rely solely on memory/training)

B9. Do you have any suggestions for how we could improve the donor eligibility screening process (e.g., speed, clarity, or accessibility)?

يمكنك كتابة الإجابة باللغة العربية أو الإنجليزية، حسب تفضيلك

Response format: Free text (paragraph)

B10. If you could design a tool to help with donor eligibility screening, what key features would it include?

يمكنك كتابة الإجابة باللغة العربية أو الإنجليزية، حسب تفضيلك

Response format: Free text (paragraph)

Part C. Post-implementation survey: Donor Eligibility Digital Assistant Evaluation

P1. How frequently have you used the Digital Donor Eligibility Assistant since its implementation?

كم مرة استخدمت المساعد الرقمي لأهلية المتبرعين؟

Response format: Single response

- ☐ Every shift
- ☐ Most shifts (>75% of the time)
- ☐ Some shifts (25–75% of the time)
- ☐ Rarely (<25% of the time)
- ☐ Never

P2. How would you rate the current donor eligibility process overall (with the new digital tool)? (matched question)

كيف تقيّم عملية تقييم أهلية المتبرعين بشكل عام (مع الأداة الرقمية الجديدة)؟

Response format: 5-point scale, single response

- ☐ 1 = Very difficult
- ☐ 2
- ☐ 3
- ☐ 4
- ☐ 5 = Very efficient

P3. On average, how long does the eligibility assessment take per donor (with the digital tool)? (matched question)

بالمتوسط، كم يستغرق تقييم الأهلية لكل متبرع باستخدام الأداة الرقمية؟

Response format: Single response

- ☐ <5 minutes
- ☐ 5–10 minutes
- ☐ 10–15 minutes
- ☐ >15 minutes
- ☐ It varies widely

P4. Which types of questions STILL cause the most uncertainty or confusion? (Select all that apply) (matched Question)

ما هي أنواع الأسئلة التي لا تزال تسبب أكثر شك أو ارتباك؟ (اختر كل ما ينطبق)

Response format: Multiple response (check all that apply)

- ☐ Medical conditions
- ☐ Medication Deferal list
- ☐ Sexual risk questions
- ☐ Travel/Malaria
- ☐ Tattoos/Hijama/Piercing
- ☐ Pregnancy/breastfeeding
- ☐ Brucellosis / raw milk
- ☐ None – tool addresses all my needs
- ☐ Other: _________

Section 3: Tool evaluation (rate your agreement with each statement)

P5. The tool is easy to use and navigate.

الأداة سهلة الاستخدام

Response format: 5-point scale, single response

- ☐ 1 = Strongly disagree
- ☐ 2
- ☐ 3
- ☐ 4
- ☐ 5 = Strongly agree

P6. The tool helps me find eligibility information quickly.

تساعدني الأداة في إيجاد معلومات الأهلية بسرعة.

Response format: 5-point scale, single response

- ☐ 1 = Strongly disagree
- ☐ 2
- ☐ 3
- ☐ 4
- ☐ 5 = Strongly agree

P7. The information in the tool is accurate and reliable.

المعلومات في الأداة دقيقة وموثوقة.

Response format: 5-point scale, single response

- ☐ 1 = Strongly disagree
- ☐ 2
- ☐ 3
- ☐ 4
- ☐ 5 = Strongly agree

P8. The tool has improved my workflow compared to before.

حسّنت الأداة سير عملي مقارنة بالسابق.

Response format: 5-point scale, single response

- ☐ 1 = Strongly disagree
- ☐ 2
- ☐ 3
- ☐ 4
- ☐ 5 = Strongly agree

P9. Overall, I am satisfied with the Digital Donor Eligibility Assistant.

بشكل عام، أنا راضٍ عن المساعد الرقمي لأهلية المتبرعين.

Response format: 5-point scale, single response

- ☐ 1 = Strongly disagree
- ☐ 2
- ☐ 3
- ☐ 4
- ☐ 5 = Strongly agree

P10. What is the ONE thing you would improve about the tool?

ما الشيء الوحيد الذي تودّ تحسينه في الأداة؟

Response format: Free text (paragraph)

P11. Any additional comments? (Optional)

أي تعليقات إضافية؟ (اختياري)

Response format: Free text (paragraph)
