# Supplementary material for: Development and Pilot Assessment of an AI-Generated Digital Donor Eligibility Screening Tool in Saudi Blood Banks: A Quality Improvement Study
Source: J Clin Med. 2026 Nov 3;15(21):8548. doi: 10.3390/jcm15218548 (PMC13764147; doi:10.3390/jcm15218548)
Supplement: Supplementary file 1 [file jcm-15-08548-s001.zip › Supplementary File S2.pdf]

Supplementary File S2

Survey response distributions for close-ended questions:

Part A. Baseline survey (n = 12)

Table S2.1. Likert-type items (1–5)

Item	Anchors (1 / 5)	Valid n	Missing	1	2	3	4	5	Median (IQR)	Mean (SD)
B1 Overall process rating	Very difficult / Very efficient	12	0	0	0	1 (8.3)	5 (41.7)	6 (50.0)	4.5 (4–5)	4.42 (0.67)
B7 Confidence in final decision	Not at all / Completely confident	12	0	0	0	1 (8.3)	4 (33.3)	7 (58.3)	5 (4–5)	4.50 (0.67)

Table S2.2. B2 — Part of the process that takes the most time (single response; valid n = 12, missing 0)

Option	n (%)
Medication deferral checks	4 (33.3)
Checking SOPs or flowcharts	4 (33.3)
Clarifying medical history	2 (16.7)
Reviewing DHQ responses	1 (8.3)
Documentation/data entry	1 (8.3)
Travel/malaria questions	0
Sexual risk questions	0
Communicating reasons for deferral	0
Other	0

Table S2.3. B3 — Average assessment time per donor (single response; valid n = 12, missing 0)

All 12 respondents selected exactly one category.

Option	n (%)
<5 minutes	0
5–10 minutes	8 (66.7)
10–15 minutes	3 (25.0)
>15 minutes	1 (8.3)
It varies widely	0

Table S2.4. B5 — Question types causing the most uncertainty (multiple response; valid n = 12, missing 0)

Option	Respondents, n (%)
Medication deferral list	9 (75.0)
Medical Conditions	9 (75.0)
Travel/malaria	4 (33.3)
Wounds/infections	2 (16.7)
Brucellosis/raw milk	2 (16.7)
Pregnancy/breastfeeding	0
Tattoos, piercing, hijama	0
Sexual risk questions	0
Other	0

Table S2.5. B6 — Frequency of consulting guidelines or supervisors (single response; valid n = 12, missing 0)

Option	n (%)
Never	0
Rarely	3 (25.0)
Sometimes	8 (66.7)
Often	1 (8.3)
Always	0

Table S2.6. B8 — Resources used most frequently (multiple response; valid n = 12, missing 0)

Option	Respondents, n (%)
Supervisor/colleague consultation	10 (83.3)
Printed SOPs/flowcharts	8 (66.7)
Internal digital database/system	4 (33.3)
Online resources (e.g., external websites)	3 (25.0)
Physical medication deferral list	3 (25.0)
None (rely on memory/training only)	0

Part B. Post-implementation survey (n = 12)

Table S2.7. P1 — Frequency of tool use since implementation (valid n =11, missing 1)

Option	n (% of 11)
Every shift	1 (9.1)
Most shifts (>75%)	0
Some shifts (25–75%)	4 (36.4)
Rarely (<25%)	6(54.5)
Never	0

Table S2.8. Likert-type items (1–5); no response was 1 or 2 on any item

Item	Anchors (1 / 5)	Valid n	Missing	3	4	5	Median (IQR)	Mean (SD)
P2 Overall process rating (with tool)	Very difficult / Very efficient	12	0	2 (16.7)	3 (25.0)	7 (58.3)	5 (4–5)	4.42 (0.79)
P5 Easy to use and navigate	Strongly disagree / Strongly agree	12	0	0	5 (41.7)	7 (58.3)	5 (4–5)	4.58 (0.51)
P6 Finds	Strongly disagree /	12	0	2 (16.7)	3 (25.0)	7 (58.3)	5 (4–5)	4.42 (0.79)

Item	Anchors (1 / 5)	Valid n	Missing	3	4	5	Median (IQR)	Mean (SD)
eligibility information quickly	Strongly agree							
P7 Information accurate and reliable	Strongly disagree / Strongly agree	12	0	2 (16.7)	3 (25.0)	7 (58.3)	5 (4–5)	4.42 (0.79)
P8 Improved workflow vs before	Strongly disagree / Strongly agree	12	0	4 (33.3)	3 (25.0)	5 (41.7)	4 (3–5)	4.08 (0.90)
P9 Overall satisfaction with the tool	Strongly disagree / Strongly agree	12	0	1 (8.3)	5 (41.7)	6 (50.0)	4.5 (4–5)	4.42 (0.67)

Table S2.9. P3 — Average assessment time per donor with the tool (single response; valid n = 12, missing 0)

Option	n (%)
<5 minutes	4 (33.3)
5–10 minutes	4 (33.3)
10–15 minutes	2 (16.7)
>15 minutes	1 (8.3)
It varies widely	1 (8.3)

Table S2.10. P4 — Question types that still cause uncertainty (multiple response; valid n = 12, missing 0; 19 selections)

Option	Respondents, n (%)
Medical conditions	7 (58.3)
Medications	6 (50.0)
Sexual risk questions	2 (16.7)
None – tool addresses all my needs	2 (16.7)
Travel/Malaria	1 (8.3)
Tattoos/Hijama/Piercing	1 (8.3)
Pregnancy/breastfeeding	0
brucellosis/raw-milk	0
Other	0
